# Biomechanical Properties and Biocompatibility of a Non-Absorbable Elastic Thread

**DOI:** 10.3390/jfb10040051

**Published:** 2019-11-16

**Authors:** Yeji Choi, Moonseok Kang, Moon Seop Choi, Jennifer Kim Song, Eugene Lih, Deahyung Lee, Hong-Hee Jung

**Affiliations:** 1 Advanced Medical Device R&D Center, HansBiomed Co. Ltd., 7, Jeongui-ro 8-gil, Songpa-gu, Seoul 05836, Korea; yjchoi@hansbiomed.com (Y.C.); eugene.lih@hansbiomed.com (E.L.); leedh@hansbiomed.com (D.L.); 2 GAROSU Plastic Surgery, Seoul 06043, Korea; flamekms@gmail.com; 3 Grace Plastic Surgery, Seoul 06524, Korea; mschoi@graceclinic.co.kr; 4 VIVA Plastic Surgery Clinic, Busan, 47285, Korea; dr.jenks@gmail.com

**Keywords:** textiles, sutures, elasticity, tensile strength, friction, materials testing

## Abstract

To date, extensive studies have been conducted to assess diverse types of sutures. But there is a paucity of data regarding biomechanical properties of commonly used suture materials. In the current experiment, we compared biomechanical properties and biocompatibility, such as tensile strength and elongation, the degree of bovine serum albumin (BSA) release, in vitro cytotoxicity and ex vivo frictional properties, between a non-absorbable elastic thread (NAT; HansBiomed Co. Ltd., Seoul, Korea) (NAT-R: NAT with a rough surface, NAT-S: NAT with a smooth surface) and the Elasticum^®^ (Korpo SRL, Genova, Italy). The degree of tensile strength and elongation of Si threads was significantly higher in both the NAT-R and -S as compared with the Elasticum^®^ (*p* < 0.05). Moreover, the degree of tensile strength and elongation of PET threads was significantly lower in both NAT-R and -S as compared with the Elasticum^®^ (*p* < 0.05). Furthermore, the degree of tensile strength and elongation of braided Si/PET threads was significantly lower in NAT-S as compared with NAT-R and Elasticum^®^ (*p* < 0.05). The degree of BSA release was significantly higher in the NAT-R as compared with Elasticum^®^ and NAT-S throughout a 2-h period in the descending order (*p* < 0.05). The degree of cell viability was significantly higher in both NAT-R and -S as compared with Elasticum^®^ (*p* < 0.05). The degree of coefficient of friction as well as the frictional force and strength was significantly higher in NAT-R as compared with NAT-S and Elasticum^®^ (*p* < 0.05). NAT had a higher degree of biomechanical properties and biocompatibility as compared with Elasticum^®^. But further experimental and clinical studies are warranted to compare the efficacy, safety, and potential role as a carrier for drug delivery between NAT and Elasticum^®^.

## 1. Introduction

Wound repair is a well-orchestrated highly-coordinated process, and it encompasses a series of phases, such as inflammation, cell proliferation, matrix deposition, and tissue remodeling [1,2]. Sutures play a role in maintaining tissue approximation, thus inducing the wound to achieve a sufficient level of tensional strength and thereby preventing wound dehiscence. Correct closure and stabilization of surgical wound margins may have a significant effect on the successful outcomes of surgery [3,4].

Sutures are classified as absorbable or non-absorbable, natural or synthetic, and multi-filament or monofilament ones [5]. Each of them has its own merits and demerits.

Non-absorbable sutures are characterized by the resistance to biodegradability. They include natural (surgical steel, silk, cotton, and linen) and synthetic non-absorbable sutures (nylon, polypropylene, and polybutester) [5,6,7]. Advantages of non-absorbable sutures include strength, a lack of premature breakage, and a minimal risk of inflammatory responses [7].

Absorbable sutures are characterized by a loss of tensile strength within 60 days with little or no tissue reaction at a predictable rate [8]. They include natural surgical gut, polygalactin (Vicryl), polyglycolic acid (Dexon), glycolic acid (Maxon), and polydioxanone (PDS). They may be preferred because they are spontaneously biodegraded [6].

Multiple factors are involved in determining the quality of tissue repair, and these include tissue characteristics, material properties of the suture, and surgical technique [9]. Therefore, selection of optimal suture material for appropriate indications will help to avoid adverse surgical outcomes [10,11,12,13].

To date, extensive studies have been conducted to assess diverse types of knots and anchor materials [14,15]. But there is a paucity of data regarding biomechanical properties of commonly used suture materials.

Elasticum^®^ (Korpo SRL, Genova, Italy) is an elastic thread with a long needle; it has recently been used for facelift procedures and its effectiveness in creating diverse facial expressions. Unlike conventional types of threads, it is extended depending on the movement of facial expression muscles. Its elasticity has been reported to contribute to producing natural lifting results [16,17].

Given the above background, we have developed a novel type of a non-absorbable elastic thread (NAT; HansBiomed Co. Ltd., Seoul, Korea). NAT is a non-absorbable elastic suture thread that consists of an elastic thread with a unilateral extension and its surrounding sheath layer. In addition, it is also composed of more than one type of elastic polymers. The surrounding sheath layer is composed of a non-elastic thread made of more than one type of non-elastic polymers. With parallel arrangement of non-elastic threads and formation of a band, the NAT is equipped with an even number (2, 4, 6, 8, 10, 12, 14 or 16) of bands braided with each other. This results in the formation of a sheath layer. We therefore conducted this experimental study to assess its biomechanical properties and biocompatibility as compared with those of Elasticum^®^.

The current article is structured as follows: Section 2 describes experimental materials and procedures. Section 3 presents the results of the experiment. Section 4 discusses them with a review of previous published studies. Section 5 draws conclusions of the current study.

## 2. Materials and Methods

### 2.1. Experimental Rationale

The current experiment was performed in accordance with the American Society for Testing and Materials (ASTM) D2256 (Standard Test Method for Tensile Properties of Yarns by the Single-Strand Method) [18]. In more detail, this method covers the measurement of tensile properties of monofilament, multifilament, and spun yarns, either single, plied, or cabled with the exception of yarns that stretch more than 5.0% when there is an increase in the tension from 0.05 to 1.0 cN/tex (0.5–1.0 gf/tex). Moreover, it also covers the measurement of the breaking force and elongation of yarns and then calculation of the breaking tenacity, initial modulus, chord modulus and breaking toughness. Guidelines for the current experiment are applicable to straight, knotted, and looped specimens. Finally, experimental conditions include those for specimens that are (1) conditioned air; (2) wet, not immersed; (3) wet, immersed; (4) oven-dried; (5) exposed to elevated temperature; or (6) exposed to low temperature [18].

### 2.2. Experimental Materials and Setting

Briefly, single-strand yarn specimens were broken on a tension testing machine at a pre-determined rate of elongation. This is followed by the measurement of the breaking force and the elongation at break. In addition, the current experiment also aimed to obtain the elongation at a specified force or the force or tenacity at a specified elongation. Furthermore, it also aimed to identify linear correlations of the density with breaking force, breaking tenacity, elongation, initial and chord modulus, and breaking toughness of the specimens [16].

For the current experiment, we defined testing variables as follows: First, tensile strength (or traction force) of the thread is a measure of the lapse of time spent during which it loses 70–80% of its initial strength. Second, elongation (or ductility of the thread) is a maximal increase in its length relative to its initial value [1,19,20].

Two types of threads were used to prepare the specimens; these include silicone (Si; Nusil Technology, Carpinteria, CA, USA) and 150 denier 48-filament high tenacity polyester (PET) yarn (Textile Development Associates Inc., Brookfield, CT, USA). Therefore, specimens were prepared using Si braided by PET. Surface properties of the specimens vary depending on the pattern of PET braiding.

**Figure 1:**
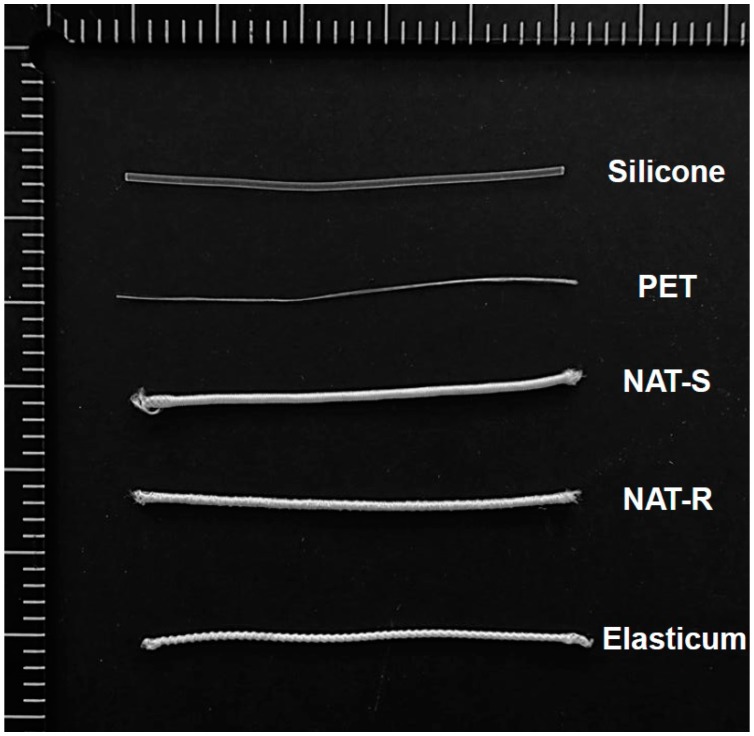
Experimental materials. Abbreviations: NAT-R, non-absorbable thread with a rough surface; NAT-S, non-absorbable thread with a smooth surface; Si, silicone; PET, polyester.

For the current experiment, the Elasticum^®^, a non-absorbable elastic suture material that is made of silicone and sheathed with PET, served as a control material [16,17]. In addition, the NAT served as a trial material. Depending on its surface properties, it is classified as NAT-R (rough surface) and -S (smooth surface). Experimental materials are summarized in Table 1. Prior to the experiment, both the control and trial materials were assessed for the measurement of dimensions that include the diameter and surface smoothness or roughness, as illustrated in Figure 1.

### 2.3. Experimental Procedures

Tensile strength was measured using an universal testing machine (UTM; Instron, Norwood, MA, USA) with a length of approximately 10 mm as the distance of the clamps along the axis of the specimens, a crosshead speed of testing of 50 mm/min, a load cell of 100 N, a room temperature of approximately 20°C, and a relative humidity of approximately 51% [21,22]. Moreover, elongation was measured as the displacement of the thread before its breakage during the measurement of its tensile strength [23].

A scanning electron microscopy (SEM) was performed to examine the ultrastructure of both the trial and control materials using the Zeiss-Merlin (Carl Zeiss Microscopy GmbH, Munich, Germany). Finally, their biocompatibility was also assessed based on a cell viability.

#### 2.3.1. Characterization of Surface Properties

**Figure 2:**
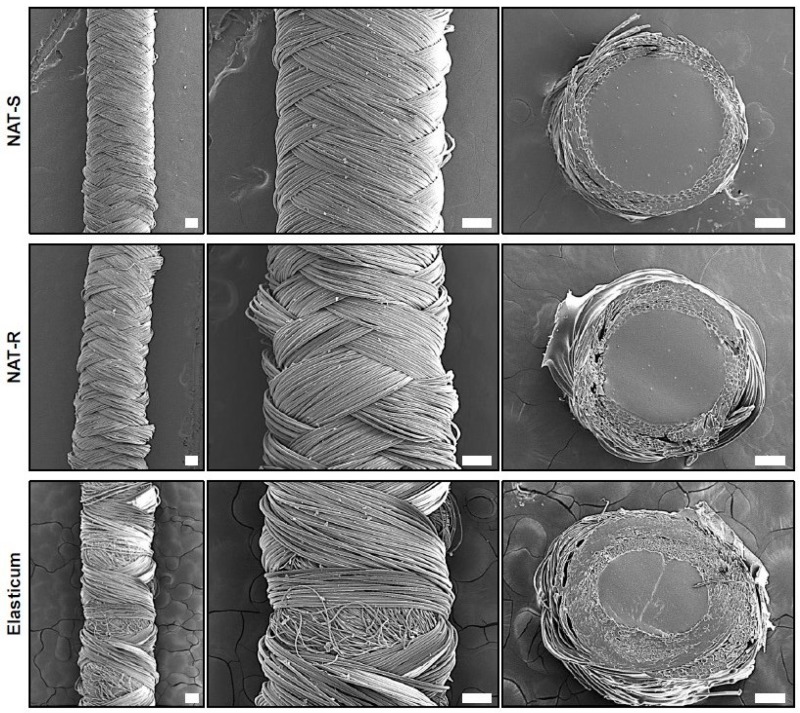
Ultrastructure of a non-absorbable thread and the Elasticum^®^. Abbreviations: NAT-R, non-absorbable thread with a rough surface; NAT-S, non-absorbable thread with a smooth surface. A field emission scanning electron microscopy of the shape and cross-section of the NAT-R and -S and the Elasticum^®^ is shown. The white bar indicates the scale of 200 µm.

Surface properties of specimens were examined on field emission scanning electron microscopy (FE-SEM). To do this, each specimen was cut at a certain size and underwent vacuum deposition. This is followed by the FE-SEM of the surface, horizontal section and morphological alterations in the surface after elongation at a magnification of 20× and 50×. On FE-SEM, both NAT-R and -S were observed to be braided in a regular weave, but the Elasticum^®^ was not (Figure 2).

#### 2.3.2. Measurement of Tensile Strength and Elongation

**Figure 3:**
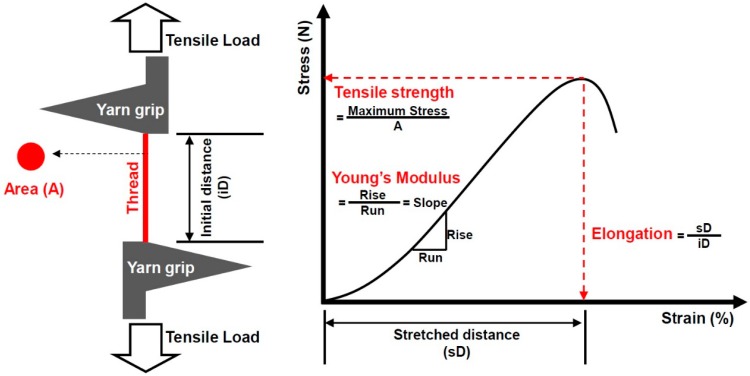
Measurement of tensile strength.

To examine the tensile properties depending on the pattern of PET braiding, tensile and elongation strength were measured using the UTM (Instron Calibration Laboratory, Norwood, MA, USA). Specimens were placed in the center of the UTM with a gap distance of 10 mm. After the fixation of specimens, the elongation speed was set at 50 mm/min and the 100 N load cell was used. All the testing values were maintained constant. Measurements of tensile and elongation strength were obtained three times and then averaged (Figure 3).

#### 2.3.3. Quantification of Release of Bovine Serum Albumin (BSA)

To measure the surface area of specimens, the release of BSA from them was quantified. First, specimens were loaded with BSA at a concentration of 2 mg/mL and then placed in a vacuum oven for 12 h. Following this, specimens were placed in a vial containing phosphate buffer saline (PBS) and then stirred in a 37 °C incubator at 70 rpm. Meanwhile, PBS was removed and then added at the same amount at certain time intervals. The mixture was treated with bicinchoninic acid (BCA) protein assay reagent (Thermo Fisher Scientific, Waltham, MA, USA) at a temperature of 37 °C for 30 min. This is followed by the measurement of absorbance using the enzyme-linked immunosorbent assay (ELISA) reader (Multiskan Sky; Thermo Fisher Scientific Inc., Waltham, MA, USA) at a wavelength of 562 nm.

#### 2.3.4. Assessment of in Vivo Stability

To assess an in vivo stability of the NAT, an in vitro cytotoxicity test was performed. L929 cells, fibroblasts derived from the murine subcutaneous tissue, were purchased from Korea Cell Line Bank (Seoul, Korea). They were cultured in Eagle’s minimum essential medium (Eagle’s MEM; Welgene Inc., Daegu, Korea) containing 10% fetal bovine serum (FBS), and were placed in a 37 °C incubator with 5% CO_2_ at a concentration of 3 × 10^4^ cells/cm^2^ for 24 h. The high-density polyethylene (HDPE) film and zinc diethyldithiocarbamate (ZDEC) polyurethane film served as the negative and positive control, respectively. Following this, specimens were eluted at a temperature of 37 °C. The resulting solution was added to L929 cells, which is followed by a 24-h additional culture. Following this, 3-(4,5-dimethylthiazol-2-yl)-2,5-diphenyltetrazolium bromide (MTT) solution (5 mg/mL stock in PBS) was placed in each well. After a 2-h culture, culture medium and MTT solution were discarded. Dimethylsulfoxide (DMSO) solution was added to each well. The well was shaken to ensure that no crystals were left. This is followed by the measurement of absorbance using the ELISA reader (Multiskan Sky; Thermo Fisher Scientific Inc., Waltham, MS, USA) at a wavelength of 570 nm.

#### 2.3.5. Measurement of the Degree of Frictional Strength within the Tissue

To assess the degree of frictional strength within the tissue, the NAT penetrated into the leg muscle of 8-week-old male Sprague Dawley ratsweighing 250 g. After complete fixation of the NAT to the UTM at a total length of 50 mm, it was left at a certain gap distance of 10 mm until it reached the grip. The elongation speed was set at 50 mm/min and the 100 N load cell was used. All the testing values were maintained constant. Measurements of the degree of frictional strength within the tissue were obtained three times and then averaged.

### 2.4. Statistical Analysis

All data was expressed as mean ± SD (SD: standard deviation). Statistical analysis was done using the SPSS 18.0 for Windows (SPSS, Chicago, IL, USA). Measurements were compared between the trial and control materials using the repeated measures analysis of variance (ANOVA) and Duncan’s post-hoc analysis. A *p*-value of <0.05 was considered statistically significant.

## 3. Results

### 3.1. Tensile Strength and Elongation

**Figure 4:**
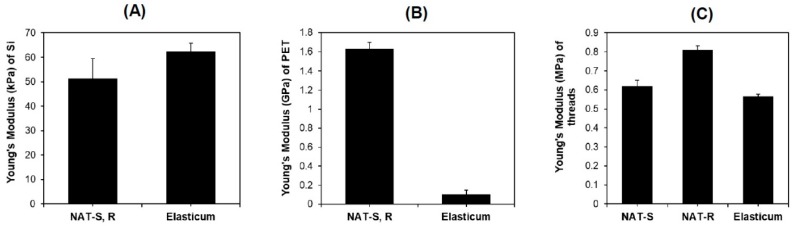
Young’s modulus. (**A**) Young’s modulus of Si was 51.39 ± 8.08 kPa in NAT and 62.40 ± 3.38 kPa in Elasticum^®^. (**B**) Young’s modulus of PET was 1.63 ± 0.07 GPa in NAT and 0.10 ± 0.05 GPa in Elasticum^®^. (**C**) Young’s modulus of braided Si/PET threads was 0.62 ± 0.03 MPa in NAT-S, 0.81 ± 0.02 MPa in NAT-R and 0.57 ± 0.01 MPa in Elasticum^®^. Abbreviations: NAT-R, non-absorbable thread with a rough surface; NAT-S, non-absorbable thread with a smooth surface; Si, silicone; PET, polyester.

Measurements of Young’s modulus were obtained, as shown in Figure 4.

Measurements of tensile strength and elongation of Si and PET are represented in Table 2.

The degree of tensile strength of Si threads was significantly higher in both the NAT-R and -S as compared with Elasticum^®^ (8.68 ± 0.41 vs. 4.84 ± 0.46, *p* < 0.05). Moreover, the degree of tensile strength of PET threads was significantly lower in both the NAT-R and -S as compared with the Elasticum^®^ (12.26 ± 0.23 vs. 23.56 ± 0.97, *p* < 0.05).

The degree of elongation of Si threads was significantly higher in both the NAT-R and -S as compared with the Elasticum^®^ (19.07 ± 0.16 vs. 14.26 ± 0.44, *p* < 0.05). Moreover, the degree of elongation of PET was significantly lower in both NAT-R and -S as compared with Elasticum^®^ (0.39 ± 0.01 vs. 1.18±0.11, *p* < 0.05).

Measurements of the degree of tensile strength and elongation of braided Si/PET threads are represented in Table 3.

The degree of tensile strength of braided Si/PET threads was significantly lower in the NAT-S as compared with NAT-R and Elasticum^®^ (43.89 ± 0.87 vs. 50.83 ± 0.89 and 49.97 ± 0.01, *p* < 0.05). Moreover, the degree of elongation of braided Si/PET threads was significantly lower in NAT-R as compared with NAT-S and Elasticum^®^ (5.53 ± 0.17 vs. 6.10 ± 0.31 and 7.16 ± 0.01, *p* < 0.05). Finally, it was significantly lower in NAT-S as compared with Elasticum^®^ (6.10 ± 0.31 vs. 7.16 ± 0.01, *p* < 0.05).

**Figure 5:**
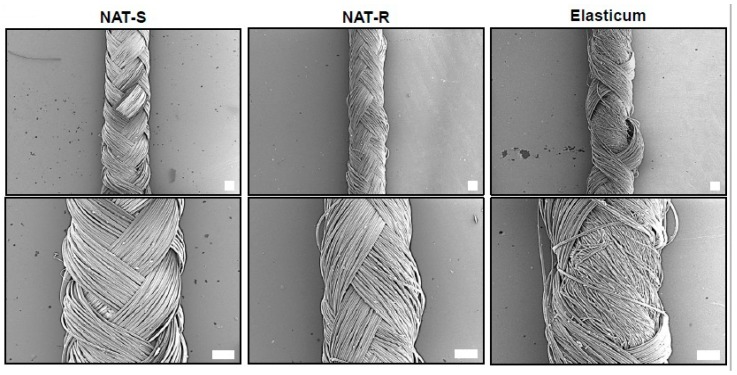
A field emission scanning electron microscopy (FE-SEM) of a non-absorbable thread and Elasticum^®^ after elongation. Abbreviations: NAT-R, non-absorbable thread with a rough surface; NAT-S, non-absorbable thread with a smooth surface.

The SEM findings of the experimental materials after elongation are shown in Figure 5. This showed that both the NAT-R and -S were observed to be braided in a regular weave.

On FE-SEM, both NAT-R and -S were observed to be braided in a regular weave, but the Elasticum^®^ was not after elongation. The white bar indicates the scale of 200 µm.

### 3.2. The Degree of BSA Release

**Figure 6:**
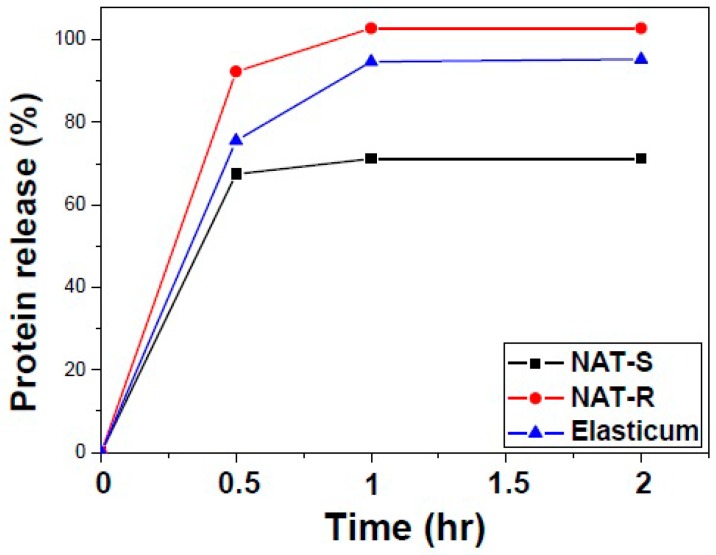
The degree of bovine serum albumin (BSA) release.

As shown in Table 4 and Figure 6, the degree of BSA release was significantly higher in NAT-R as compared with Elasticum^®^ and NAT-S throughout a 2-h period in the descending order (*p* < 0.05). Of note, there were no further changes in the degree of BSA release at 1 and 2 h in both NAT-R and -S but there was a time-dependent increase in it in Elasticum^®^.

### 3.3. In Vitro Cytotoxicity

**Figure 7:**
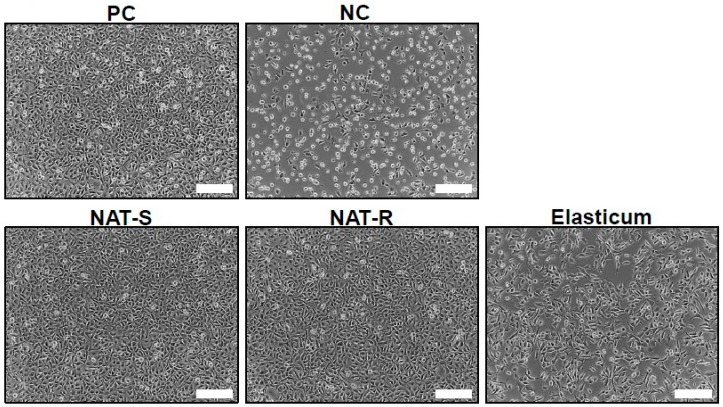
In vitro cytotoxicity. Abbreviations: NAT-R, non-absorbable thread with a rough surface; NAT-S, non-absorbable thread with a smooth surface; PC, positive control; NC, negative control.

As shown in Table 5, there was no significant difference in the degree of cell viability between NAT-R and -S (*p* > 0.05). But it was significantly higher in both NAT-R and -S as compared with Elasticum^®^ (*p* < 0.05). An in vitro cytotoxicity test showed that the percentage of apoptotic L929 cells was notable in Elasticum^®^ (Figure 7).

### 3.4. Ex Vivo Frictional Properties

As shown in Table 6, the degree of coefficient of friction as well as the frictional force and strength was significantly higher in NAT-R as compared with NAT-S and Elasticum^®^ (*p* < 0.05). But there was no significant difference in it between NAT-S and Elasticum^®^ (*p* > 0.05).

## 4. Discussion

Selection of optimal sutures for tissue repair is dependent on multiple factors, such as its caliber, properties of the target tissue (e.g., fascia, tendon or bone), the rigidity and elasticity of fixation (e.g., fracture fixation or tendon repair), superficial or deep location of the repair, and biocompatibility or biodegradability [24,25]. Moreover, their clinical applicability is closely associated with diverse factors, such as their biomechanical properties, characteristics of the target tissue, and the degree of potential biodegradability [26,27,28].

To date, several studies have been conducted to assess the biomechanical properties of sutures [15,29,30,31,32,33]. We also assessed the biomechanical properties of the NAT as compared with those of Elasticum^®^ in the current experiment.

A sufficient level of biomechanical characteristics and properties, such as excellent tensile strength, dimensional stability, lack of memory, knot security, and flexibility to prevent damages to the tissue are requirements of the suture [23]. There is a time-dependent increase in the strength and adherence of the sutured tissue; there is a significant increase in the strength of the flap between 1 and 2 weeks postoperatively. Use of sutures with a poor strength may cause its untimely breakage, eventually interfering with tissue repair [1].

Elongation-at-break of sutures is closely associated with reorientation of filaments in the direction of the axis of braiding [34]. Monofilaments and braided structures are the two key elements constituting sutures. Monofilament sutures are equipped with a high degree of stiffness. Likewise, braided structures with a rough surface are vulnerable to breakage despite their flexibility [35]. Saber et al. noted that the variation of braiding angle had a great effect on elongation of sutures. These authors also noted that knot slippage is another key determinant of success of sutures [36]. But we failed to consider braiding angle and knot slippage in the current study, which deserves further experiments.

The NAT is equipped with an adequate level of tensile and elastic properties, an appropriate level of flexibility and resistance to traction, which is essential for not only suturing, ligating, fixing, and lifting the skin and soft tissue at surgical sites but also being used as a drug delivery system (DDS). Although conventional types of DDS have no specificity, it has a specific effect in controlling the process of drug delivery [37]. If incorporated in drugs, it would be used to deliver them to internal tissues or organs for which direct delivery of them is very difficult [38]. Thus, drug-eluting NAT may be developed, which is based on the rationale that polymeric controlled release systems elevate local concentrations of drugs without causing excessive systemic levels. This enables the NAT to deliver active pharmaceutical ingredients, such as non-steroidal anti-inflammatory drugs (NSAIDs), antibiotics, and a variety of growth factors (e.g., fibroblast growth factor [FGF], vascular endothelial growth factor [VEGF], transforming growth factor-β [TGF-β], ephrins and epidermal growth factor [EGF]), during surgical procedures without placing foreign body materials which might delay the wound healing process or cause infections in the wound bed [39,40,41]. Indeed, diverse natural and synthetic polymers have been explored as potential carriers for drug delivery [42,43,44]. Of these, biodegradable synthetic polymers are known to have a great potential as a carrier for drug delivery [43]. They require a vehicle, such as poly(d,l-lactide-co-glycolide) (PLGA), encapsulating proteins, inhibiting biodegradation, and promoting in vivo activity and providing controlled release. The vehicle is used to encapsulate and release numerous model and recombinant proteins, such as BSA [45,46,47]. In the current experiment, we measured the release of BSA from the NAT, which is based on a previous published study showing a correlation between the in vitro release of BSA and the degradation rate of the polymer [48].

## 5. Conclusions

To summarize, our results are as follows:

1. The degree of tensile strength and elongation of Si threads was significantly higher in both NAT-R and -S as compared with Elasticum^®^ (*p* < 0.05). Moreover, the degree of tensile strength and elongation of PET threads was significantly lower in both NAT-R and -S as compared with Elasticum^®^ (*p* < 0.05). Furthermore, the degree of tensile strength and elongation of braided Si/PET threads was significantly lower in NAT-S as compared with NAT-R and Elasticum^®^ (*p* < 0.05).
2. The degree of BSA release was significantly higher in NAT-R as compared with Elasticum^®^ and NAT-S throughout a 2-h period in the descending order (*p* < 0.05).
3. The degree of cell viability was significantly higher in both NAT-R and -S as compared with Elasticum^®^ (*p* < 0.05).
4. The degree of coefficient of friction as well as the frictional force and strength was significantly higher in NAT-R as compared with NAT-S and Elasticum^®^ (*p* < 0.05).

Based on our results, it can be concluded that both NAT-R and -S had a higher degree of biomechanical properties and biocompatibility as compared with Elasticum^®^. But further studies are warranted to compare the clinical applicability, efficacy, and safety between them. Moreover, the potential role of NAT as a carrier for drug delivery deserves more attention.

## Figures and Tables

**Table 1:** Experimental materials.

	Si		PET		Diameter(mm)
	Diameter (mm)	EA	Diameter (mm)	EA	
NAT-R	1.00	1	0.05	8	1.20
NAT-S	1.00	1	0.05	8	1.20
Elasticum^®^	0.52	2	0.20	2	1.10

Abbreviations: NAT-R, non-absorbable thread with a rough surface; NAT-S, non-absorbable thread with a smooth surface; Si, silicone; PET, polyester.

**Table 2:** Tensile strength and elongation of silicone (Si) and polyester (PET) in a non-absorbable thread (NAT) and Elasticum^®^.

Variables	Values			
	NAT		Elasticum^®^	
	Si	PET	Si	PET
Tensile strength (N)	8.68 ± 0.41 *	12.26 ± 0.23 **	4.84 ± 0.46 *	23.56 ± 0.97 **
Elongation (mm/mm)	19.07 ± 0.16 *	0.39 ± 0.01 **	14.26 ± 0.44 *	1.18 ± 0.11 **

* Statistical significance at *p* < 0.05, ** Statistical significance at *p* < 0.05.

**Table 3:** Tensile strength and elongation of a non-absorbable thread (NAT) and Elasticum^®^.

Variables	Values		
	NAT-S	NAT-R	Elasticum^®^
Tensile strength (N)	43.89 ± 0.87 ^ab^	50.83 ± 0.89 ^a^	49.97 ± 0.01 ^b^
Elongation (mm/mm)	6.10 ± 0.31 ^a^	5.53 ± 0.17 ^bc^	7.16 ± 0.01 ^abc^

Abbreviations: NAT-R, non-absorbable thread with a rough surface; NAT-S, non-absorbable thread with a smooth surface. Different letters indicate statistical significance at *p* < 0.05.

**Table 4:** The degree of bovine serum albumin (BSA) release.

Experimental Materials	Time Points			
	0 h	0.5 h	1.0 h	2.0 h
NAT-R	0	92.31	102.83	102.83
NAT-S	0	67.50	71.16	71.16
Elasticum^®^	0	75.61	94.71	95.29

Abbreviations: NAT-R, non-absorbable thread with a rough surface; NAT-S, non-absorbable thread with a smooth surface.

**Table 5:** The degree of cell viability.

Variables	Values		
	NAT-R	NAT-S	Elasticum^®^
Cell viability (%)	72.19 ± 5.39 ^a^	71.67 ± 0.68 ^b^	55.52 ± 2.91 ^ab^

Abbreviations: NAT-R, non-absorbable thread with a rough surface; NAT-S, non-absorbable thread with a smooth surface. Different letters indicate statistical significance at *p* < 0.05.

**Table 6:** Ex vivo frictional properties.

Variables	Values		
	NAT-R	NAT-S	Elasticum^®^
Frictional force(N)	0.62 ± 0.21 ^ab^	0.37 ± 0.14 ^b^	0.35 ± 0.12 ^a^
Frictional strength (MPa)	0.55 ± 0.18 ^ab^	0.32 ± 0.12 ^a^	0.31 ± 0.11 ^b^
Coefficient of friction	0.30 ± 0.08 ^ab^	0.15 ± 0.05 ^a^	0.15 ± 0.04 ^b^

Abbreviations: NAT-R, non-absorbable thread with a rough surface; NAT-S, non-absorbable thread with a smooth surface. Different letters indicate statistical significance at *p* < 0.05.
